# Can diagnostic injections predict the outcome in foot and ankle arthrodesis?

**DOI:** 10.1186/1471-2474-15-11

**Published:** 2014-01-09

**Authors:** Mark Stegeman, Berbke To Josephine van Ginneken, Bastiaan Boetes, Mirjam Tuinhout, Jan Willem Karel Louwerens, Bartele Alexander Swierstra

**Affiliations:** 1 Department of Orthopaedics and Rheumatology, Maartenskliniek Woerden, Woerden, The Netherlands; 2 Department of Orthopaedics, Maartenskliniek Woerden, Woerden, The Netherlands; 3 Department of Orthopaedics, IJsselland Ziekenhuis, Capelle aan den IJssel, The Netherlands; 4 Department of Orthopaedics, Sint Maartenskliniek Nijmegen, Nijmegen, The Netherlands

**Keywords:** Foot pain, Foot function, Arthrodesis, Diagnostics, Fluoroscopically guided anesthetic injection

## Abstract

**Background:**

Intra-articular anesthetic drug injections are claimed to confirm the localization of the pain in order to treat the pain. The aim of the present study was to evaluate whether a positive effect of injection could be indicative for a successful outcome of future arthrodesis.

**Methods:**

74 Patients underwent fluoroscopically guided and contrast confirmed anesthetic joint injections for diagnostic reasons. Before and after injection, pain was measured by use of the Visual Analogue Scale (VAS) in rest and after exercise. Pain reduction was expressed as delta VAS (dVAS). Also, the Foot Function Index (FFI) was obtained. Based on the effect of the diagnostic injection and various clinical factors, patients were advised a conservative treatment (conservative group, n = 34) or an arthrodesis of the affected joint (operative group, n = 40). After a median follow-up period of 3.6 years (range 2.1 to 4.3 years) patients were again invited to complete the FFI and VAS in rest and after exercise. For data-analysis purposes the patients were assigned to four different groups, based on the result of injection and the occurrence of surgery. Wilcoxon signed rank tests and Mann Whitney U tests were used for statistical analysis.

**Results:**

Based on the analysis of the four groups we found that surgery, irrespective of the presence of pain reduction after injection, was related to improvement of VAS and FFI. Patients with conservative treatment always showed worse VAS and FFI scores, even when previous injections showed an improvement of VAS.

**Conclusions:**

Fluoroscopically-guided anesthetic injections of the supposed painful foot-ankle joint seem not to be indicative for a successful outcome of an arthrodesis of the affected joint. However, the sole occurrence of surgery shows a significant difference in VAS and FFI scores, where conservative treatment does not. The local hospital review board granted permission for this study. Ethical approval was not required for this study.

## Background

The precise treatment of foot and ankle pain depends on accurate assessment with regard to the cause and the site of origin of the pain. However, this assessment, accurately identifying the source of pain, can be difficult in the complex hindfoot with its numerous joints and ligaments [1-4]. Most often a diagnosis is based on the medical history, a careful physical examination and one or more imaging modalities. However, the changes or absence of changes seen on these imaging modalities may not correlate with the extent or alleged localization of the clinical symptoms. This can be due to various causes like the presence of multilevel pathology [3,5]. In case of multilevel pathology it can be unclear whether pain is coming from one joint or more joints, or whether it is caused by problems involving the soft tissues.

Intra-articular fluoroscopically-guided anesthetic drug injections may help to confirm the alleged localization of the source of pain by differentiating between two separate joints and between intra-and extra-articular origin of pain, and thus may help in predicting therapeutic outcome. This technique has been described as an aid to the diagnosis of shoulder pain, chronic wrist pain, referred pain in the upper limb and in the diagnosis of nerve entrapment syndromes [6-9]. This procedure is also known for differentiation of hip from spinal problems [1].

We evaluated whether pain reduction after intra-articular anesthetic drug injection could be indicative for a successful outcome (i.e. pain and function) of arthrodesis or conservative treatment in foot and ankle pathology. Our hypothesis states that pain reduction after intra-articular anesthesia could be indicative for a successful outcome of future arthrodesis.

## Methods

Between September 2002 and December 2004, 99 patients (> 18 years of age) underwent a diagnostic fluoroscopically-guided joint injection. Out of these 99 patients 25 patients were excluded from this study for the following reasons. Six patients who after surgery developed a non-union, thus compromising the outcome by other means than a correct or not correct indication for surgery were excluded. Five patients with contrast leakage, two with CRPS, and four patients with additional surgery were also excluded. Finally, eight patients were lost to follow-up. In patients with bilateral injections only the first injection was included [10]. Thus, 74 patients participated in the study. All patients had experienced foot complaints for more than 12 months and the majority of patients at the time of referral to our department had already been treated conservatively. Conservative treatment consisted mostly of modification of shoe wear or inlays. Pre-operative diagnoses included 39 patients with posttraumatic arthritis, 31 patients with osteoarthritis and 4 patients with rheumatoid arthritis.

All affected joints were injected by a skeletal oriented radiologist under fluoroscopic control (Philips BV300, The Netherlands) using an antiseptic technique and a standardized protocol [4,11]. Confirmation of the intra-articular position of the needle was performed by use of 0.5-1 ml of contrast material (Omnipac 300, GE Healthcare, UK or Xenetix 300, Guerbet Group The Netherlands). The used anesthetic drug consisted of bupivacaine (Actavis Group, The Netherlands) 0.25% and citanest 1% (AstraZeneca BV, The Netherlands). Depending on the size and capacity of the joint the volume anesthetic drug ranged from 1.5 to 6 ml. Special attention was given to leakage of the fluid to connecting joint.

A Visual Analogue Score measuring pain (VAS) was obtained closely before and 30 minutes after the injection in rest and after exercise [12]. Foot Function Index (FFI) was obtained before the injection [13-15]. Two foot and ankle surgeons (JWL and BS) decided whether conservative or operative treatment was advised to a patient. This advise was based on history, physical examination and additional information acquired through X-ray examination, CT scans and furthermore a substantial difference in VAS scores before and after the injections. Based on the literature [2,12] and clinical experience a dVAS of 3 or more was determined as the minimum effect of the diagnostic injection in order to regard this as a positive parameter to advise surgery. Subjective criteria such as the involvement in law suits, workers compensation issues, and pain behavior were taken into account also.

Four groups were distinguished and analyzed in this study:

Group 1: Preoperative successful anesthetic injection resulting in successful surgery (34 patients). In this group there was a positive result on injection resulting in a VAS decrease of at least 3 points. Based on the effect of the diagnostic injection and various clinical factors the doctor would advise surgery and the patient agreed to have surgery. The VAS postoperative was at least three points lower than preoperatively. This group seems to show an association between positive result on injection and success after surgery.

Group 2: Preoperative successful anesthetic injection and refusal to surgery (19 patients). This group consists of patients who had a successful preoperative anesthetic injection with a VAS decrease of at least 3 points and choose not to have surgery although they were advised to. No significant improvement was seen from conservative treatment in this group.

Group 3: Preoperative unsuccessful anesthetic injection but surgery anyway (six patients).

In this group the unsuccessful injection did not deter the doctor or the patient from surgical intervention. These patients did actually gain from the surgery as their VAS decreased more than three points, a significant decrease. This would argue against our hypothesis that preoperative anesthetic injection would predict the result of surgery.

Group 4: Preoperative unsuccessful anesthetic injection and no surgery (15 patients). This is the group in which the negative result to anesthetic injection resulted in the decision not to operate. No significant improvement was seen from conservative treatment in this group.

After a mean follow-up of 3.6 years (range 2.1 to 4.3 years), all patients were requested to complete a VAS for pain in rest and after exercise and the FFI. The conservative group was asked if they had undergone surgery elsewhere during the follow-up period and if they were using any kind of foot/ankle orthotics or shoe adaptations. When complications such as non union occur after surgery the VAS for pain and FFI measurements are gravely influenced and it was decided not to include these patients in the study to prevent a bias. Thus these patients were excluded from the study as mentioned earlier.

Wilcoxon signed rank tests and Mann Whitney U tests were used for statistical analysis. We evaluated whether pain reduction after intra-articular anesthetic drug injection could be indicative for a successful outcome (i.e. pain and function) of arthrodesis or conservative treatment in foot and ankle pathology. The local hospital review board granted permission for this study. Ethical approval was not required for this study.

## Results

Table 1 depicts the injected joint locations and number of different hind-and midfoot joints. Most joints included are hindfoot joints, comparable in size and surface. Table 2 shows the patient characteristics of the four groups. The male to female ratio in all groups was equal, with the exception of group 3. The age for all groups is similar. Tables 3, 4, 5, 6 show the differences in VAS and FFI before and after the treatment for the four different groups. There was a significant improvement in VAS at rest in patients with a significant improvement after the injection and after surgery (Table 3). Patients without improvement on injection, but who still underwent surgery showed a trend towards improvement after surgery. Patients without surgery (with and without improvement on injection) showed no statistical difference in VAS scores. Table 4, depicting the differences in VAS scores during exercise before and after treatment showed similar results. The decrease in VAS after surgery is more clear during exercise for the patient groups who underwent surgery. Table 5 and Table 6 show FFI disability scores and activity limitation scores respectively for the different groups. Both surgery groups showed clear improvement where the no surgery groups showed no improvement.

**Table 1:** Injected joint locations

**Injected joint**	**Number of patients**
Talocrural	9
Subtalar	37
Talonavicular	12
Calcaneocuboid	3
Naviculocuneiform	4
First tarsometatarsal	5
Second tarsometatarsal	2
Third tarsometatarsal	2

**Table 2:** Patient Characteristics

	**Gender** ♂**/**♀^**a**^	**Age mean +/- SD**
**Total group**	36♂/38♀	45,6 +/- 14,4
**Group I**		
**Pos-injection + surgery**	15♂/19♀	43,3 +/- 14,9
**Group II**		
**Pos-injection – surgery**	9♂/10♀	48,9 +/- 15,8
**Group III**		
**Neg-injection + surgery**	5♂/1♀	50,8 +/- 7,3
**Group IV**		
**Neg-injection – surgery**	7♂/8♀	44,7 +/- 13,4

^a^♂/♀ indicating males/females.

**Table 3:** VAS pain scores in rest before and after treatment

	**Baseline VAS rest +/- SD**	**VAS rest at follow-up +/- SD**	**P-value**
**Group I**			
**Pos-injection + surgery**	3,5 +/- 2,4	1,3 +/- 1,4	<.001*
**Group II**			
**Pos-injection–surgery**	3,1 +/- 2,1	3,5 +/- 1,8	.345
**Group III**			
**Neg-injection + surgery**	1,8 +/- 1,7	0,4 +/- 0,7	.068^†^
**Group IV**			
**Neg-injection–surgery**	3,8 +/- 2,8	3,0 +/- 1,4	.289

*Indicating a significant improvement (p < 0.05) from baseline to follow-up.

^**†**^Indicating a trend to improvement (p < .10) from baseline to follow-up.

**Table 4:** VAS pain scores during exercise before and after treatment

	**Baseline VAS rest +/- SD**	**VAS rest at follow-up +/- SD**	**P-value**
**Group I**			
**Pos-injection + surgery**	7,2 +/- 1,5	2,5 +/- 1,51,5	<.001*
**Group II**			
**Pos-injection–surgery**	6,3 +/- 1,8	5,4 +/- 2,5	.247
**Group III**			
**Neg-injection + surgery**	6,0 +/- 2,3	2,8 +/- 1,3	.046*
**Group IV**			
**Neg-injection–surgery**	6,0 +/- 2,3	5,8 +/- 2,5	.694

*Indicating a significant improvement (p < 0.05) from baseline to follow-up.

**Table 5:** FFIb foot function disability scores before and after treatment

	**Baseline FFIb +/- SD**	**FFIb at follow-up +/- SD**	**P-value**
**Group I**			
**Pos-injection + surgery**	51,5 +/- 22,9	17,6 +/- 13,6	<.001*
**Group II**			
**Pos-injection–surgery**	44,7 +/- 13,9	43,4 +/- 14,8	.845
**Group III**			
**Neg-injection + surgery**	53,3 +/- 20,0	17,8 +/- 18,9	.028*
**Group IV**			
**Neg-injection–surgery**	42,2 +/- 14,8	39,7 +/- 17,9	.826

*Indicating a significant improvement (p < 0.05) from baseline to follow-up.

**Table 6:** FFIc foot function activity limitation scores before and after treatment

	**FFIc +/- SD**	**FFIc at follow-up +/- SD**	**P-value**
**Group I**			
**Pos-injection + surgery**	49,2 +/- 22,5	20,2 +/- 16,1	<.001*
**Group II**			
**Pos-injection–surgery**	44,4 +/- 19,9	38,6 +/- 18,3	.230
**Group III**			
**Neg-injection + surgery**	56,5+/- 22,5	16,0 +/- 19,9	.028*
**Group IV**			
**Neg-injection–surgery**	33,7 +/- 24,3	30,1 +/- 22,1	.925

*Indicating a significant improvement (p < 0.05) from baseline to follow-up.

## Discussion

The aim of the present study was to evaluate whether a positive effect of injection could be indicative for a successful outcome of future arthrodesis. The results indicate that arthrodesis has a positive effect on pain and function, irrespective of pain reduction after injection. We found that only the intervention of surgery has a predictive value for the relief of pain and improvement of function, and the effect on injection is not related to the outcome of either surgery or conservative treatment. The only way to make this observation possible, is that by coincidence the patients who postponed or chose not to have surgery even with a positive effect on injection formed a very interesting control group, unforeseen by the investigators. Also the group of patients without effect on injection but with surgery formed a small but interesting control group and proved the hypothesis to be false. In our goal to investigate the predictive value of anesthetic injection on the outcome after arthrodesis of the hindfoot or midfoot the injection was defined as an important, but not the only, criteria to either perform surgery or not. A plausible explanation is that the clinical view of the orthopedic surgeon ultimately determines best whether a positive result after surgery is to be expected.

The results of our study are in contrast to the conclusion of various authors mentioning a correlation between a positive result on anesthetic injection and successful outcome after surgery [4,7,16]. Previous studies have described the use of anesthetic drug injections in the foot and subtalar joints [1-5,17-19]. A retrospective study conducted by Khoury et al. described in a group of 20 patients a positive correlation between the effect of foot joint injections and subsequent effect of an arthrodesis [2]. The studies of Crawford, Ruhoy and Bell all conclude that a correlation is present between a positive reaction on injection and a positive result after arthrodesis, but there was no mention of a control group [4,7,16]. We suspect that in these studies the same assumption is made as our initial idea that a positive reaction to injection correlates with a good result of surgery, but when control groups would be included the assumption that injections are predictive of good effect on arthrodesis could be rejected. In these articles there were no patients mentioned who did not have a good result on injection and did not have surgery so the correlation between successful injection and good outcome was believed to be unbiased while it is possible that only the surgical intervention itself was the important variable. Our techniques for injection and exclusion of patients with contrast leakage is consistent with other studies [1,3].

Our study does have limitations. The design of the study was primarily focused on the predictive value of injections, but the surgeons also focused on X rays, CT scans, physical examination and questionnaires in advising the patient surgery or conservative treatment. Also the role of the surgeon is a confounding factor because the decision for operative or conservative treatment was made with full knowledge of the result of the injection. The cutoff point of VAS 3 was chosen based on publications by DeLoach [12] stating that every VAS measurement is 20 mm imprecise and the publication by Khoury [2] stating that 65% and 50% pain relief after intra articular injection served as a measure to perform arthrodesis. A relatively large number of patients was excluded or lost to follow up (25 of 99 patients). Finally, the use of VAS scores was originally meant for chronic pain measurement, whereas for acute postoperative pain or post injection pain measurement is of less value [12].

For future research we suggest the regular workup for hindfoot arthrodesis followed by a decision to operate or not. The intra articular anesthetic injection should be performed in all cases blinded to the surgeon and the patient. When a true correlation between injection and outcome after surgery or after conservative treatment is found, we can decide whether anesthetic injections are a diagnostic tool of value or not. An example of a good study design is presented in the study of Lucas as the focus in this study has been on the level of confidence of the surgeon before and after a blinded intra articular injection [18]. The injection altered the surgical plan in 82% of 50 patients. Unfortunately the result of the surgical or conservative treatment remains unclear in this study, so the conclusion that the injections aid in the outcome for the patient cannot be drawn. Finally, to investigate the value of an anesthetic injection in the clinical decision making, one should not only study the relationship between a positive test and a significant improvement of the surgery but also take into account the clinically meaningful improvement in a patient’s quality of life by determining the Minimum Clinical Important Difference.

In the range of additional diagnostic techniques either MRI, CT or diagnostic injections are favored [4,18,20]. Because recently contrast enhanced ultrasound guided injections have been found to significantly increase the accuracy of injections in the foot, this would be an interesting sequel to this study [21,22].

## Conclusions

Pain reduction after intra articular injections seems not to be indicative for successful outcome after arthrodesis in the foot. This finding contradicts with many published articles on this subject. We found that a careful history, thorough physical examination and radiology imaging will provide the necessary information to recommend surgery and combined with informed consent from the patient predict a good outcome.

## Abbreviations

VAS: Visual analogue scale; FFI: Foot function index.

## Competing interests

The authors declare that they have no competing interest.

## Authors’ contributions

MS participated in the design and coordination of the study and drafted the manuscript, BvG performed the statistical analysis and helped to draft the manuscript, BB participated in the design, collected data and helped revising the manuscript, MT participated in the design, collected data and helped revising the manuscript, JL participated in the design and helped revising the manuscript, BS participated in the design and coordination of the study and helped revising the manuscript. All authors read and approved the final manuscript.

## Pre-publication history

The pre-publication history for this paper can be accessed here:

http://www.biomedcentral.com/1471-2474/15/11/prepub
